# Leveraging pleiotropy for the improved treatment of psychiatric disorders

**DOI:** 10.1038/s41380-024-02771-7

**Published:** 2024-10-10

**Authors:** Damian J. Woodward, Jackson G. Thorp, Christel M. Middeldorp, Wọlé Akóṣílè, Eske M. Derks, Zachary F. Gerring

**Affiliations:** 1 Brain and Mental Health, QIMR Berghofer Medical Research Institute, Brisbane, QLD Australia; 2 School of Biomedical Science, Queensland University of Technology, Brisbane, QLD Australia; 3 School of Biomedical Sciences, Faculty of Medicine, University of Queensland, Brisbane, QLD Australia; 4 Department of Child and Adolescent Psychiatry and Psychology, Amsterdam UMC, Amsterdam Reproduction and Development Research Institute, Amsterdam Public Health Research Institute, Amsterdam, The Netherlands; 5 Arkin Mental Health Care, Amsterdam, The Netherlands; 6 Levvel, Academic Center for Child and Adolescent Psychiatry, Amsterdam, The Netherlands; 7 Child Health Research Centre, University of Queensland, Brisbane, QLD Australia; 8 Child and Youth Mental Health Service, Children’s Health Queensland Hospital and Health Service, Brisbane, QLD Australia; 9 Greater Brisbane Clinical School, Faculty of Medicine, University of Queensland, Brisbane, QLD Australia; 10 Healthy Development and Ageing, Walter and Eliza Hall Institute of Medical Research, Melbourne, Victoria, Australia

**Keywords:** Psychiatric disorders, Genetics, Drug discovery

## Abstract

Over 90% of drug candidates fail in clinical trials, while it takes 10–15 years and one billion US dollars to develop a single successful drug. Drug development is more challenging for psychiatric disorders, where disease comorbidity and complex symptom profiles obscure the identification of causal mechanisms for therapeutic intervention. One promising approach for determining more suitable drug candidates in clinical trials is integrating human genetic data into the selection process. Genome-wide association studies have identified thousands of replicable risk loci for psychiatric disorders, and sophisticated statistical tools are increasingly effective at using these data to pinpoint likely causal genes. These studies have also uncovered shared or pleiotropic genetic risk factors underlying comorbid psychiatric disorders. In this article, we argue that leveraging pleiotropic effects will provide opportunities to discover novel drug targets and identify more effective treatments for psychiatric disorders by targeting a common mechanism rather than treating each disease separately.

## Introduction

There are significant challenges in identifying and developing new medications for human diseases. Less than 10% of drugs that begin phase I clinical trials are estimated to gain regulatory approval [1, 2]. Failure rates are notably worse in psychiatry, with the field having the second lowest likelihood of approval at 6.2% (the lowest being oncology). There are several reasons why a clinical trial might fail, such as safety, commercial, or operational factors. However, a significant contributor to trial failure is due to a lack of efficacy, particularly in the later stages of drug development [3].

An approach to increase trial success rates involves incorporating human genetic data to reveal novel biological insights [4]. Studies have demonstrated that drug compounds are more likely to be approved if their genetic targets were previously identified by human genetic evidence, with the likelihood increasing if exact causal genes are known [4, 5]. Large-scale genome-wide association studies (GWAS) have identified hundreds of replicable genetic risk factors for complex diseases, including candidate causal genes [6]. These studies can establish the genetic evidence (i.e., interactions between drugs and disease-associated genes) needed for improved drug discovery and treatment [7].

The time and cost associated with traditional drug development pipelines are a significant concern in the pharmaceutical industry [8]. An alternative method to identify novel drugs for psychiatric disorders is the use of existing drugs for a new clinical indication(s), known as drug repurposing [9]. This process can bypass some de novo drug discovery steps by identifying an already approved or investigational drug that may have been abandoned due to lack of efficacy yet passed safety assessment [10]. Due to existing research on drug safety and pharmacokinetics in humans, drug repurposing can decrease the timeframe and cost of de novo drug development with a lower risk of failure.

**Fig. 1:**
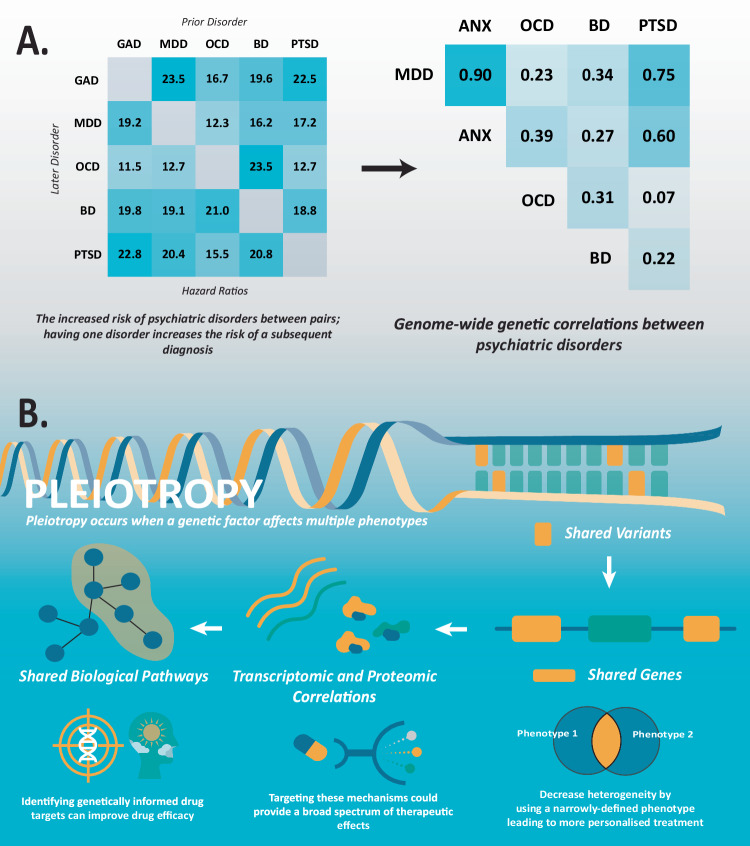
Leveraging pleiotropy in psychiatric disorders to improve treatment outcomes. **A** The increased risk of psychiatric disorder diagnosis from a prior disorder reflects widespread genetic correlations. **B** Targeting pleiotropic mechanisms for the treatment of multiple conditions. Hazard ratios between comorbidity pairs were obtained from McGrath et al. [12]. Genetic correlations adapted from Derks et al. [7]. Acronyms: generalised anxiety disorder (GAD), anxiety disorders (ANX), major depressive disorder (MDD), obsessive-compulsive disorder (OCD), bipolar disorder (BD), post-traumatic stress disorder (PTSD).

Psychiatric disorders co-occur in the same individual more often than expected by chance (known as comorbidity) (Fig. 1A) [11, 12]. Large positive genetic correlations are also frequently observed between pairs of psychiatric disorders [7]. One explanation behind these genetic correlations is pleiotropy [13–15], which occurs when a genetic factor is associated with multiple phenotypes (Fig. 1B). Investigating these shared genetic risk factors may uncover potential aetiological overlap between disorders for drug targeting.

Here, we will argue that shifting drug development towards targeting pleiotropic mechanisms is a promising avenue for determining effective drug targets in psychiatry. A robust pleiotropic mechanism can be identified using GWAS summary statistics and appropriate statistical methods. By integrating transcriptomic, proteomic, and drug datasets, these mechanisms can elucidate shared downstream molecular effects for drug repurposing opportunities. Further network medicine approaches can identify additional drug targets and reveal latent connections between disorders or between disorders and drug compounds.

Using pleiotropy for drug target identification has several potential benefits. Psychiatric disorders are highly diverse in their aetiology, symptoms, onset, and course, which limits our understanding of specific disease mechanisms and the development of personalised medicine [7, 16]. Narrowing down drug target identification to more homogenous phenotypes, such as co-occurring symptoms or conditions, can restrict this heterogeneity and improve power to distinguish the underlying mechanisms. Additionally, a single drug could treat multiple conditions by targeting a shared mechanism to provide a broader therapeutic impact.

This review will show how leveraging psychiatric pleiotropy can be an encouraging approach to identifying more effective drug targets. Methods and research investigating the nature of pleiotropy in psychiatric disorders and strategies to identify and prioritise drug candidates for repurposing will be examined.

## Understanding pleiotropy in psychiatric disorders

There is significant genetic overlap between psychiatric disorders. Family and twin studies first identified positive correlations and patterns of inheritance between psychiatric disorders [17–19]. The introduction of methods for estimating heritability and genetic correlations using GWAS summary statistics, such as linkage disequilibrium score regression (LDSC) [20] later identified genetic correlations across numerous psychiatric phenotypes [15, 21, 22]. Genetic correlations for psychiatric disorders range between –0.17 for ADHD and OCD to 0.9 for depression and anxiety [7]. Disentangling the pleiotropic effects that underlie these correlations is a key challenge of psychiatric genetics.

**Fig. 2:**
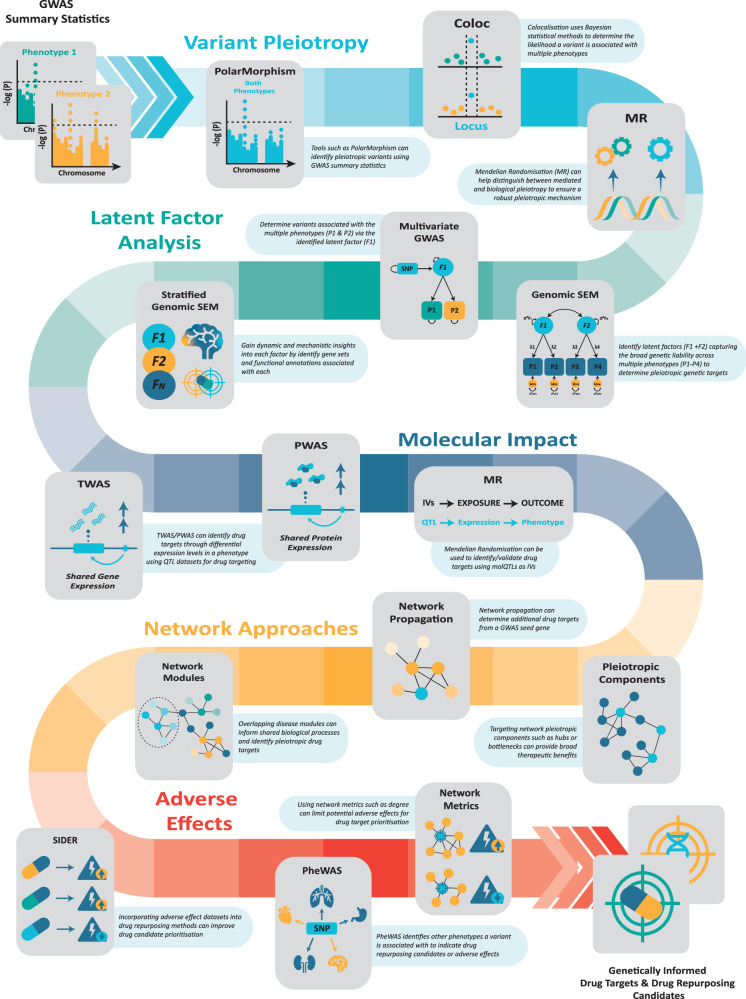
Overview of methods for leveraging pleiotropy in psychiatric disorders using genome-wide association studies (GWAS) to identify genetically informed drug targets and drug repurposing candidates. Acronyms: Colocalisation (Coloc), mendelian randomisation (MR), genomic structural equation modelling (genomic SEM), genome-wide association study (GWAS), transcriptome-wide association study (TWAS), protein-wide association study (PWAS), quantitative trait loci (QTL), instrumental variables (IVs), phenome-wide association study (PheWAS).

Pleiotropy can be studied across different levels. Firstly, pleiotropy is observed at a regional or loci level, with a comprehensive study analysing eight psychiatric disorders identifying 146 loci associated with a psychiatric phenotype, with 109 displaying cross-phenotype associations [23]. Secondly, transcriptome and proteome studies have shown extensive correlations in (imputed) gene expression levels [24] and shared causal and interacting proteins [25], demonstrating pleiotropy at a molecular level. Finally, the downstream biological effects of pleiotropy can be elucidated at a network and pathway level. A study identified 49 pathways associated with three psychiatric traits, which clustered into processes involving histone, synaptic biology, neuronal, and immune pathways [26]. Figure 2 outlines methodologies investigating pleiotropy at these various levels to be integrated for drug repurposing.

Targeting pleiotropic mechanisms may advance transdiagnostic treatments in psychiatry, i.e., using a single treatment for symptoms or conditions across multiple diagnoses. Evidence in clinical practice shows that psychiatric transdiagnostic treatments are an already recognised concept, e.g., selective serotonin reuptake inhibitors for the treatment of major depressive disorder and generalised anxiety disorder [27]. As most drug targets are proteins [28], improving our understanding of the shared molecular factors and pathways between disorders can further transdiagnostic treatments by targeting the potential shared consequence of genetic variation.

## Identifying variant-level pleiotropy between disorders

A cross-phenotype association is a statistical discovery of a single locus displaying an association with multiple phenotypes. Identifying a shared genetic variant/s between comorbid disorders from this association can help us understand the molecular causes underlying psychiatric comorbidity. This, in turn, provides opportunities for therapeutic interventions that target the molecular consequence of a shared causal variant rather than treating the conditions separately. However, several considerations are needed to establish a pleiotropic mechanism from a cross-phenotype association.

### Initial considerations

#### Cross-phenotype associations

The first consideration is using appropriate statistical methods to discover cross-phenotype associations. Numerous analytic approaches exist for the joint evaluation of multiple traits (see [29] for a full review). Briefly, method choice depends on data availability, phenotype distribution, number of traits, and if there are overlapping samples between datasets. While methods that jointly analyse traits directly are typically more powerful [29], they are substantially limited in scope by requiring individual-level genotype data and all traits to be measured in the same individuals. These conditions can be challenging to obtain due to privacy and data restrictions and many traits going unmeasured (e.g., low prevalence disorders). More recently, methods have been developed that require only univariate GWAS summary statistics and account for sample overlap, e.g., LDSC [20], MTAG [30] and genomic SEM [31]. This has enabled the identification of cross-phenotype associations at a larger scale and across a wide range of traits, which can be performed at various levels, from genome-wide to regional or a variant level.

#### Pleiotropic mechanism

**Fig. 3:**
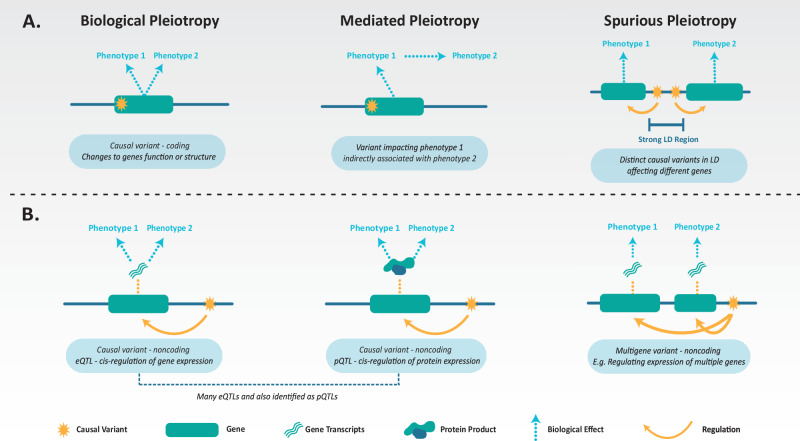
Types of pleiotropic mechanisms underpinning psychiatric cross-phenotype associations. **A** The three divisions of pleiotropy. Biological: when a variant is associated with multiple phenotypes owing to true biological effects. Mediated: a phenotype causally influences another phenotype, resulting in a variant to be indirectly associated with the second phenotype. Spurious: when a variant is identified as pleiotropic due to sources of bias, linkage disequilibrium (LD) patterns, or study design artefact. **B** Additional types of biological pleiotropic mechanisms. Causal noncoding variants in *cis*, regulate gene and protein expression (single and multigene) with biological products affecting multiple phenotypes. Acronyms: expression quantitative trait loci (eQTLs), protein quantitative trait loci (pQTLs).

The second consideration is whether the variant has an actual biological effect on each phenotype. This uncertainty stems from the different mechanisms of pleiotropy that underly cross-phenotype associations. These mechanisms include biological, mediated, and spurious pleiotropy (Fig. 3A) [32]. Biological pleiotropy (horizontal pleiotropy) is when a variant affects two or more phenotypes through a direct biological effect. Biological pleiotropy can be broken down further into single-gene pleiotropy (the variant impacts one gene that affects two or more phenotypes) and multi-gene pleiotropy (the variant impacts multiple genes affecting multiple phenotypes) [13]. Figure 3B demonstrates biological pleiotropy via pleiotropic noncoding variants associated with gene/protein expression changes. Mediated pleiotropy (or vertical pleiotropy) arises when one phenotype causally affects a second phenotype, resulting in a variant associated with phenotype one being indirectly associated with phenotype two. Finally, spurious pleiotropy involves variants associated with multiple phenotypes from sources of bias, including phenotype misclassifications, study design artefacts, or strong LD patterns.

### Methodological approaches

When identifying pleiotropic variants from GWAS summary statistics, taking the intersection of variants associated with each trait is less optimal than methods that directly model pleiotropic relationships, as distinguishing pleiotropic variants is limited by the power of both GWAS to detect the variant.

Several resources are available to address this issue and estimate the probability of a pleiotropic variant/s across phenotypes [33–37]. For example, PolarMorphism can assess two or more phenotypes by converting trait-specific effect sizes into polar coordinates to evaluate variant sharedness while distinguishing biological pleiotropic variants from mediated variants [33]. Another method, PLACO, has been shown to identify an equal amount of pleiotropic variants as PolarMorphism; however, it can only examine two traits at a time [34]. Colocalisation is another approach using Bayesian statistical methods to estimate the probability that a single variant in a shared locus is causal in both GWAS [38]. Other colocalisation methods have been developed to examine more than two traits simultaneously and have different assumptions regarding the number of causal variants in a locus [37, 39–41].

The improved accessibility of phenotypic data from large-scale biobanks has enabled researchers to combine phenotypic and genetic data to identify pleiotropic variants. Phenome-wide association studies (PheWAS) analyse variants or groups of variants against a diverse set of phenotypes [42]. This method identifies variant associations with multiple diseases, thereby identifying potential pleiotropic effects, although the underlying mechanism behind these associations still needs to be clarified. PheWAS is highly versatile, demonstrating use in validating drug targets [43], studying risk variants associated with polygenic risk scores [44], uncovering pleiotropic biological function [45], predicting drug adverse effects [46], and drug repurposing [47].

### Variant pleiotropy and drug repurposing

The mechanism underlying a pleiotropic variant can inform therapeutic interventions. If a drug target is associated with two disorders via mediated pleiotropy, treating one disorder would either alleviate or exacerbate the symptoms of the second disorder, depending on their relationship. Additionally, targeting a mediated pleiotropic mechanism would only benefit the second disorder if the first disorder is present. Targeting a biological pleiotropic mechanism can help or worsen each disorder or benefit one disorder and worsen the other, depending on the variant direction of effect. As it is likely that a combination of these mechanisms is contributing to psychiatric comorbidity, investigating these various models is required to distinguish robust drug targets.

A variant’s effect direction has implications in treating multiple disorders. A drug compound targeting shared mechanisms with opposite effects may help one phenotype but inadvertently negatively influence the other. Additionally, variant direction influences the ideal drug mechanism. For example, a gain-of-function variant would increase the function of its downstream effectors, thus requiring an inhibitor or antagonist for effective treatment. A recent method, genetic priority scores, prioritises genetic targets and drug indications derived from an integration of genetic evidence [48]. Genetic priority scores also incorporate the direction of genetic effect to determine the drug mechanism for repurposing opportunities.

### Limitations and challenges

The biggest challenge when identifying a robust pleiotropic variant for drug repurposing is discerning the underlying mechanisms. One approach, Mendelian Randomisation (MR), can help distinguish between mediated and biological pleiotropy. MR is a frequently used method for inferring causation using genetic associations from GWAS data [49]. The foundation of MR relies on Mendel’s laws of segregation and independent assortment, where variants are inherited randomly. This method utilises variants as instrumental variables (IVs) to enable a practical approach for causal inference between an exposure variable and an outcome phenotype (if key assumptions are satisfied). The assumptions of MR include 1) the IV is strongly associated with the exposure, 2) the IV is not associated with confounders between exposure and outcome, and 3) the IV is only associated with the outcome through the exposure.

In addition to determining causal exposure-outcome relationships, MR can serve as a test for mediated pleiotropy [13]. Notably, due to psychiatric disorders’ unknown biology, bidirectional relationships, heterogeneity, and high levels of pleiotropy and polygenicity, thorough sensitivity analyses and caution in interpretations are essential [50]. In light of these limitations, several methodologies are available to clarify these complex relationships and rule out alternative hypotheses [50, 51]. Other mediation analyses have been created to break down the total effect of the variant on a phenotype into direct and indirect effects [29, 52]. Another tool, BUHMBOX, can identify whether observed associations between two diseases are caused by biological pleiotropy or subgroup heterogeneity in disease cases, determining potential spurious pleiotropy [53].

Another challenge in determining a pleiotropic variant is caused by linkage disequilibrium (LD). A variant identified by GWAS may not be truly causal but is in linkage with the actual causal variant. Fine-mapping resources use Bayesian frameworks to prioritise variants by integrating LD data, functional information, and association patterns to estimate the posterior probability of each variant being causal [54, 55]. fastPAINTOR is an additional method that aims to increase fine-mapping accuracy at pleiotropic risk loci by leveraging pleiotropy to strengthen the causal variant signal [56]. Using fine mapping to distinguish the likely causal variant affecting multiple phenotypes can ensure robust downstream drug targets.

## Modelling higher-order factors of psychopathology

Certain psychiatric diseases co-occur more frequently than others and group together at clinical and subclinical levels [11, 23]. Similarly, modelling of the genetic correlations between disorders has revealed that subsets of disorders cluster together (e.g. internalising disorders or compulsive disorders) [57], suggesting the presence of a higher-order structure of psychopathology at a genetic level. Shifting focus from individual disorders toward these higher-order components can reveal key pleiotropic mechanisms for therapeutic intervention.

Multivariate approaches can identify higher-order dimensions of broad liability, known as latent factors, that explain the observed genetic or phenotypic variance between multiple traits. These higher-order latent factors are not directly measured but inferred using various statistical methods such as exploratory factor analysis [58] or structural equation modelling (SEM) [59]. The identification of latent factors of genetic liability across disorders can inform the aetiological structure of psychopathology and subsequently inform pleiotropic mechanisms for drug targets.

### Initial considerations

Latent Factor Structure

Several transdiagnostic latent factor structures have been proposed to model the comorbidity patterns of psychiatric disorders identified using phenotypic, twin or family data. Key examples include a single p factor model [60] that captures the general psychopathology across all disorders, a bi-factor model [58, 61] that divides traits by internalising vs externalising symptoms and behaviours, or a three-factor model including internalising, externalising, and a thought disorder factor [60]. The p factor is a frequent feature of dimensional approaches and may reflect the overall pleiotropic biology underlying psychiatric risk. However, breaking down the *genomic* p factor into intermediate components to capture more specific (i.e., less heterogeneous) disease clusters may be more beneficial for biological insight [21]. A recently developed psychiatric classification system, the Hierarchical Taxonomy of Psychopathology (HiTOP) [62], proposes a hierarchical system to capture different levels of specificity, including higher-order dimensions of psychopathology, which can account for pleiotropy/comorbidity between disorders directly [7]. The discovery of pleiotropy drug targets can be improved by incorporating these higher-dimensional frameworks.

### Methodological approaches

**Fig. 4:**
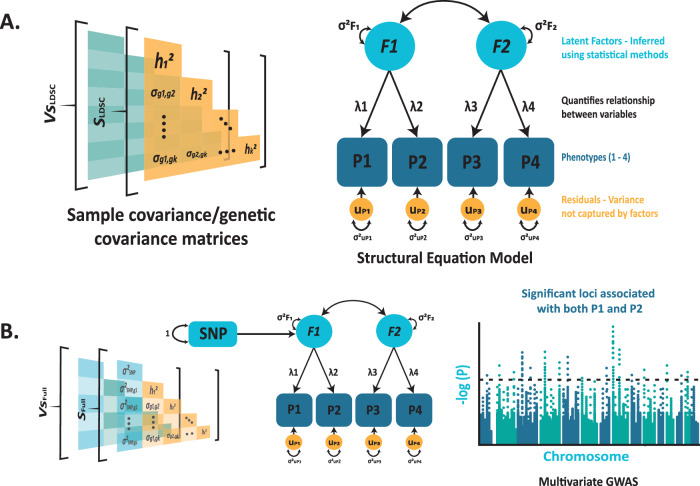
Using structural equation modelling to identify shared and unique liability between phenotypes. **A** Example of a two-factor, four phenotype model fitted to the sample covariance/genetic covariance matrices calculated by linkage disequilibrium score regression (LDSC). Factor 1 (F1) captures the broad liability between phenotypes 1 and 2 (P1, P2). Factor 2 (F2) captures phenotype 3 and 4 (P3-4). **B** Identifying significant variants (SNPs) associated with a latent factor. Example demonstrates adding SNP effect to the matrices to estimate a given SNPs effect on factor 1. Multivariate genome-wide association study (GWAS) identifies loci operating through the factor, i.e., associated with P1 and P2.

*Genomic* SEM analyses the joint genetic architecture of numerous phenotypes using only GWAS summary statistics (Fig. 4A) [31]. Genomic SEM has been used to identify a four-factor model capturing genetic overlap between 11 major psychiatric disorders, including compulsive, psychotic, neurodevelopmental, and internalising factors [21]. An additional substance-use factor has been determined using a common factor model of genetic liability between four substance-use disorders [63]. These disease factors could provide a promising starting point for identifying pleiotropic drug targets within these clusters.

*Genome-wide* SEM is another multivariate method that uses phenotypic covariance to construct an SEM, incorporating raw genotype data to identify variants associated with the modelled factors [64, 65]. This method models phenotypic with genetic information; however, it can be limited by the accessibility of raw genotype data and what phenotypic information is available across all samples.

Pleiotropic decomposition regression (PDR) is an alternative approach to decomposing the covariance of multiple traits into factors or components [66]. This method deviates from other decomposition methods by modelling the distribution of variant effect sizes to separate variants into components they affect the most, thereby limiting the number of components a variant is associated with. It is argued that this is more mechanistically informative, as knowing the traits an individual SNP affects informs a single mechanism or component [66]. PDR is limited by the number of traits that can be examined simultaneously due to computational demands and does not produce standard errors.

### Latent modelling for pleiotropy and drug repurposing

Identifying genetic latent factor models can provide insight into shared aetiology and identify drug targets for treating multiple disorders. An application of genomic SEM, *multivariate* GWAS, can determine variants impacting disorders through the inferred factors (Fig. 4B) [31]. Another addition, *stratified* genomic SEM, identifies gene sets and functional categories that disproportionately contribute to shared genetic risk, revealing mechanistic and dynamic insights between disease clusters [21]. The downstream effects on shared molecular mechanisms and pathways influencing lower-order disorders can be determined by identifying these variants or gene sets associated with disorder clusters or pairs.

Transcriptomic data can be integrated with genomic SEM to examine gene expression patterns associated with genetically overlapping psychiatric disorders, known as transcriptome-wide structural equation modelling (T-SEM) [67]. Grotzinger et al. [67] used T-SEM to identify 466 genes with expression levels associated with a five-factor model across 13 major psychiatric disorders. Additionally, the study investigated existing drug compounds to target the shared risk pathways, identifying 35 drug-gene pairs for the thought disorders factor disorder, one for the internalising disorder factor, and five for the p factor [67]. The results suggest these drugs have a transdiagnostic effect targeting the psychiatric disorders clustered within these factors.

### Limitations and challenges

Latent factor analysis has many diverse analytical applications; however, several limitations exist. Primarily, the limitations of the statistical analysis apply to the method used, i.e., the same limitations as SEM apply to genomic SEM. Some notable limitations of SEM and genomic SEM include:

1. SEM relies on the user to specify the initial model of relationships between latent variables and the observed variables. Bias estimates and inaccurate conclusions can occur if the model is misspecified or lacks justification.
2. Various models may fit the data with similar effectiveness or better, and determining the one perfect model is not practical [68]. A model fitting the data too well can risk overfitting, reducing generalisability performance, decreasing model robustness, and introduce noise.
3. Including traits with extreme correlations (i.e., highly multicollinear traits) will introduce a bias to a particular model owing to their substantial genetic overlap [69].
4. Genomic SEM only uses summary-level data, limiting the investigation of phenotypic or environmental causal pathways.
5. The power to detect a genetic drug target is determined by the power of the inputted GWAS in a given model, as seen in [67], which identified the majority of drug-gene interactions with well-powered GWAS (i.e., thought disorders).

## Elucidating molecular consequences for drug targeting

The identification of pleiotropic variants for psychiatric disorders may provide new prospects for the development of drugs that target a shared biological mechanism. To characterise the potential biological effects of a pleiotropic variant, we must integrate diverse functional genomic data from different cellular contexts. The recent availability of omics data from various tissues and cell types and improvements in integrative, systems biology-based approaches offer opportunities to establish the functional consequences of DNA sequence variation.

### Initial considerations

#### Data type

The type of molecular data is the first consideration when integrating functional data for drug repurposing. GWAS-identified variants are enriched in noncoding regions [70] and are believed to have an intermediate effect on disease via various regulatory mechanisms. These effects can be examined by integrating molecular quantitative trait loci (molQTL) datasets, with these variants associated with variation in molecular traits. Expression quantitative trait loci (eQTL) are a type of molQTL associated with changes in gene expression. eQTLs can be further divided into *cis*-eQTLs, variants associated with the expression of nearby genes, or *trans*-eQTLs, variants associated with changes in gene expression from a further distance or a different chromosome. Compared to *cis*-eQTLs, *trans*-eQTLs typically have smaller effects and thus require larger samples to detect and tend to be more tissue-specific [71]. Another molQTL, protein quantitative trait loci (pQTL), are variants associated with changes in protein expression. Many pQTLs are often recognised as eQTLs, with one study estimating 61% of pQTLs also identified as eQTLs via data obtained from post-mortem brain samples [72].

Using gene expression levels (eQTLs) and protein abundance (pQTLs) to determine genetic targets for therapeutics have their strengths and weaknesses (see Table 1). The primary argument is whilst eQTLs capture the more immediate molecular effects of disease-associated variants, variation in the post-translational stages is more therapeutically actionable, and these do not always correlate with each other [73]. Incorporating both data layers would be preferable for a comprehensive understanding of a variant’s functional consequence and provide robust evidence for any identified drug targets.

**Table 1:** Comparing expression quantitative trait loci (eQTLs) and protein quantitative trait loci (pQTLs) for drug repurposing in psychiatry.

	Cost	Ease of Measurement	Brain Dataset Availability	Proximity to Genetic Variation^a^	Epitope Artefact^b^	Therapeutically Actionable
**eQTLs**	Lower	Higher	Higher	More proximal	No	Less actionable
**pQTLs**	Higher	Lower	Lower	More distal	Yes	More actionable

^a^Gene expression is a more proximal consequence of genetic variation and is less likely to be influenced by other factors in comparison.

^b^*cis*-pQTLs may be in linkage with a non-synonymous variant that modifies the protein binding epitope, resulting in a false positive association.

#### Dataset availability

The second consideration for the functional interpretation of a pleiotropic variant is dataset availability. molQTL will differ between tissues, cell types, developmental stages, and environmental stimuli. Therefore, incorporating disease-relevant tissue data and exploring molQTLs across varying contexts when possible is essential. For psychiatric disorders, there are several publicly available brain eQTL datasets, including GTEx, a resource of eQTLs across 13 brain tissues collected from post-mortem donors [74], PsychENCODE a large dataset of 1,866 individuals [75], and MetaBrain, a meta-analysis conducted on 14 brain datasets (including GTEx and PsychENCODE) [76]. While less advanced than RNA sequencing, profiling the human proteome has made progress, resulting in pQTL identification in blood tissue [77] or brain tissue [78].

Conventional eQTL resources utilise bulk tissue expression samples, restricting insights into specific cell types, development stages, and cell states. A promising new approach is using single-cell transcriptomic assays to analyse dynamic contexts that bulk expression cannot capture by calculating gene expression at a single-cell resolution. Studies analysing brain cell type-specific eQTLs have demonstrated that many affected genes have differing expression levels in various cell types [76, 79]. While single-cell datasets are less available, significant advances have been made. Some examples of available datasets include the development stages of the human prefrontal cortex [80], brain cell types [79], and databases/portals sharing single-cell findings, e.g., scQTLbase [81] or Single Cell Portal [82].

### Methodological approaches

There are three primary methods to incorporate molQTL to identify functional mechanisms from GWAS data. The first, transcriptome-wide association studies (TWAS), uses *cis*-eQTL datasets to impute gene expression from genetic variants via multivariate models [9, 83]. This method generates genetically regulated expression prediction models by modelling eQTL information. These models predict gene expression from GWAS summary statistics to indicate a gene’s expression level and association with the trait of interest, i.e., if a gene is upregulated or downregulated in the phenotype. Reflecting TWAS, proteome-wide association studies (PWAS) can integrate pQTL and GWAS data to impute protein expression and identify potential drug-repurposing targets by utilising the same tools available from TWAS [83, 84].

**Fig. 5:**
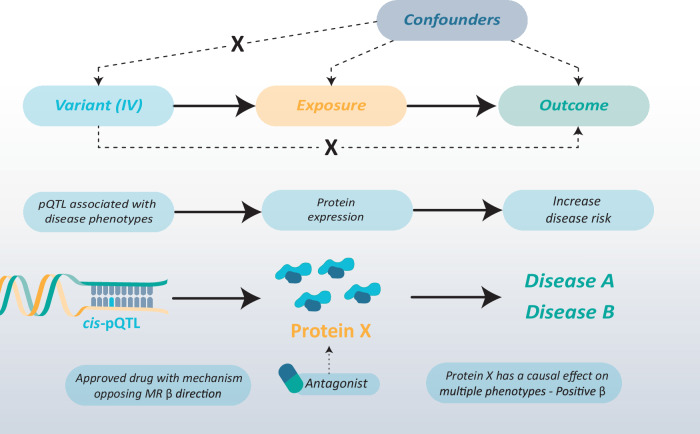
Mendelian Randomisation for drug target validation using molecular expression data. Credibly causal molecular quantitative trait loci (molQTLs) as instrumental variables (IVs) to validate that the expression of the target is causal with disease phenotype (outcome). If a drug's mechanism opposes the direction of the estimated exposure-outcome effect (β), an approved drug is prioritised for repurposing. The example illustrates that this protein X expression increases disease risk; therefore, a drug with a mechanism opposing the target, e.g., an antagonist, can be a repurposing candidate.

The second method, Mendelian Randomisation (MR), also integrates molQTL and GWAS data. As outlined previously, MR is typically used for causal inference of an exposure to an outcome using genetic variants as instrumental variables (IVs). MR, for the application of drug repurposing, utilises *cis* variants associated with targetable proteins as IVs, i.e., pQTLs or eQTLs [85]. These IVs are used for drug repurposing based on a drug compound having therapeutic potential if its protein target (the exposure) causally impacts an outcome in the right direction as the intended pharmacological mechanism (Fig. 5). As proteins are proximal effectors of biological processes, it has been argued that there is less chance to break the no horizontal pleiotropy assumption compared to downstream exposures [86]. Additional MR approaches, such as multi-response MR [87] or two-stage multivariate MR [88], enable the modelling of exposures on multiple outcomes (e.g., two comorbid phenotypes) rather than performing MR for each outcome independently.

Colocalisation is the third method and is frequently used with TWAS/PWAS/MR to further prioritise genes or proteins most likely to influence disease [38, 84, 89]. As previously discussed, colocalisation can identify pleiotropic variants across multiple phenotypes. However, it can also include molecular phenotypes through molQTL datasets. Applying this method with TWAS/PWAS can identify putatively causal genes or proteins associated with multiple diseases via changes in expression. Many other approaches have expanded upon MR and TWAS methods to improve accuracy or address limitations (Table 2).

**Table 2:** Methods building on Transcriptome-wide association studies (TWAS) and Mendelian Randomisation (MR).

Method	Description	Reference
**Causal-TWAS (cTWAS)**	Fine-mapping approach for identification of GWAS causal genes by adjusting for genetic confounders to reduce false positives	[124]
**Context-specific genetics (CONTENT)**	Decomposes gene expression of individuals into context-specific and context-shared components to generate predictors of gene expression for each component	[125]
**Fine mapping of causal gene sets (FOCUS)**	A fine-mapping approach for gene-trait association signals from TWAS, accounting for LD and prediction weights, to determine sets of genes likely to contain the causal gene	[126]
**Fine-mapping of gene sets (FOGS)**	Fine mapping of gene sets in a locus to prioritise putative causal genes by accounting for local LD and improve statistical power	[127]
**Gene-based integrative fine-mapping through conditional TWAS (GIFT)**	Fine-map causal genes by controlling the predicted expression of all other genes in proximity, limiting LD confounding and false positives	[128]
**Heterogeneity in dependent instrument (HEIDI)**	Often used in conjunction with SMR to help distinguish pleiotropy from linkage, i.e., the variant is causal for both phenotype and gene expression	[94]
**Joint tissue imputation (JTI)**	Leverages genetic and expression data from multiple tissues or cell types for gene association discovery. It also incorporates MR into JTI for causal inference (MR-JTI)	[129]
**Multi-response MR**	Incorporates multiple outcomes in an MR framework to identify exposures that affect several or individual outcomes	[87]
**Multivariable MR**	Estimates the causal relationships of multiple exposures on an outcome using numerous genetic variants	[130]
**Probabilistic Mendelian Randomisation (PMR-Egger)**	Unifies TWAS and MR into a single framework to test for putatively causal genes in the presence of horizontal pleiotropy	[131]
**Summary-based Mendelian Randomisation (SMR)**	Integrates GWAS summary statistics with expression data to identify genes associated with a phenotype via changes in expression.	[94]
**Transcriptome-wide Mendelian Randomisation (TWMR)**	A multivariate MR method using expression as exposures to estimate the causal effect of gene expression on the phenotype	[132]
**Two-stage multivariate MR (MRMO)**	A multivariate analysis to jointly model the causal effects of an exposure on multiple outcomes	[88]

These methods can be integrated to improve the identification of putatively causal genetic targets for drug repurposing.

### Molecular pleiotropy and drug repurposing

Integrating functional and genetic data can improve our understanding of the shared aetiological causes between comorbid phenotypes. This combination can identify pleiotropic genes related to specific biological processes. For instance, Antón-Galindo et al. [90] determined which genes from dopaminergic and serotonergic processes were significantly associated with multiple or individual addiction-related phenotypes through predicted expression levels [90].

Drug repurposing candidates can be prioritised using functional data to provide direction for the pleiotropic targets. Firstly, a drug can be prioritised if it directly modulates a disease genetic target in the opposite direction to its expression, e.g., an antagonist drug targeting an overexpressed gene. Secondly, signature mapping is a computational method that compares the disease gene expression signature to a drug-gene expression signature [91]. A signature describes the differential gene expression levels from perturbation caused by a drug compound or disease. This method prioritises drug compounds opposing the disease signature to theoretically normalise gene expression levels.

Many studies have identified drug repurposing candidates in psychiatric disorders based on genetically regulated expression levels in human brain tissue using the described methods (see Table 3). Developing upon this idea, leveraging pleiotropic variants or latent factors to discover genetically regulated gene or protein expression targets could identify transdiagnostic treatments for psychiatric disorders. Hatoum et al. [63] integrated multiple methodologies, including signature mapping, latent factor analysis, and TWAS, to identify 104 drug-repurposing candidates targeting an addiction latent factor. These drugs are predicted to reverse the addiction disease-expression profile across multiple lower-order conditions [63].

**Table 3:** Studies using molQTL datasets for identifying drug targets and repurposing opportunities with genome-wide association studies of psychiatric disorders.

Authors	Year	molQTL Used	Key molQTL Method	Additional Analysis	Drug Targets/Drug Repurposing Key Findings
Baird et al. [133]	2021	eQTLs	Two-Sample MR	Colocalisation, MR-PheWAS	Identified 23 drug targets for SCZ and one target for anorexia, BD, and MDD
Fabbri et al. [134]	2021	eQTLs	TWAS	Signature Mapping	Identified drug repurposing candidates for MDD subtypes - muscarinic receptor antagonist (treatment resistance MDD), heat shock protein inhibitors (anxious MDD), metabolism modulators (weight gain MDD)
Gasper et al. [135]	2019	eQTLs	TWAS	MAGMA, Drug Targetor	24 druggable genetic targets for MDD, with enrichment in drug groups: monoamine reuptake inhibitors, sex hormones, antipsychotics, and antihistamines
Gedik et al. [136]	2023	eQTLs, pQTLs, mQTLs	SMR	HEIDI	Vitamin D and omega-3 supplementation may be beneficial for BD
Greco et al. [89]	2023	eQTLs, pQTLs	TWAS/PWAS	Colocalisation	*PDE4B* as a genetically informed target for CUD*HYAL1* increased protein expression plausibly increases the risk for CUD
Grotzinger et al. [67]	2023	eQTLs	TWAS	T-SEM	Determined drugs targeting gene expression changes for thought disorder factor (35 drug-gene pairs), internalising disorder factor (1 drug-gene pair) and *p* factor (5 drug-gene pair)
Hatoum et al. [63]	2023	eQTLs	TWAS	Genomic SEM, Signature Mapping, PheWAS	104 approved medications opposing the transcriptional profile of the identified addiction factor
Jiang et al. [137]	2023	eQTLs	SMR	Signature Mapping, HEIDI	Identified potential mechanisms for the anti-depressive effects of statins and demonstrated shared pharmacological effects between statins and antidepressants
Koch et al. [138]	2022	eQTLs	TWAS	Network Propagation, Signature Mapping	Four drugs with significant opposition to disease-expression signature to treat core ASD symptoms
Li et al. [139]	2023	eQTLs, pQTLs	Two-Sample MR	Colocalisation	Identified targets using eQTLs – 31 SCZ, 7 BD, 2 MDD, 1 ADHD; using pQTLs – 5 SCZ, 2 BD, 1 ADHD
Li et al. [140]	2023	eQTLs, pQTLs	TWAS/PWAS	Colocalisation	Prioritised four candidate causal genes for anxiety disorder as potential therapeutic targets
Liu et al. [141]	2023	eQTLs, pQTLs	Two-Sample MR	PPI network and pathways	25 targets across SCZ, BD, MDD, and ADHD using eQTLs, and 10 targets across SCZ, BD, and MDD using pQTLs. 2 targets overlapped.
Liu et al. [142]	2021	eQTLS, pQTLs	TWAS/PWAS	Colocalisation	61 genes with genetically regulated protein levels associated with four psychiatric disorders. 18 overlapped with genetically regulated transcriptome levels
Lu et al. [85]	2023	pQTLs	Two-Sample MR	Colocalisation	9 circulating protein-to-disease associations as potential targets across seven phenotypes
Reay et al. [84]	2022	eQTLs, pQTLs	TWAS/PWAS	Two-Sample MR, Colocalisation, PES	Five targets for SCZ and one target for BD using transcriptomic and proteomic data.
Rodriguez-Lopez et al. [143]	2020	eQTLs	TWAS	WGCNA, Predicted Expression PRS	Co-expression module with druggable hub genes associated with schizophrenia
Wingo et al. [144]	2021	pQTLs	PWAS	TWAS, SMR, HEIDI, PPI network and pathways	19 putatively causal gene targets for depression via brain protein modulation
Wingo et al. [145]	2022	pQTLs	PWAS	Colocalisation, TWAS, SMR, HEIDI, PPI Networks	11 genes associated with PTSD via brain protein abundance, with 9 showing high confidence.
Woodward et al. [146]	2023	eQTLs	TWAS	MAGMA, Colocalisation, Signature Mapping	64 putative causal genes associated with anxiety with three novel drug groups demonstrating potential therapeutic effect
Zhou et al. [147]	2023	eQTLs	TWAS	MAGMA, Signature Mapping	Six medications predicted to reverse the transcriptional profile of problematic alcohol use

Molecular quantitative trait loci (molQTL), Expression quantitative trait loci (eQTLs), Protein quantitative trait loci (pQTLs), Methylation quantitative trait loci (mQTLs), Transcriptome-wide association study (TWAS), Protein-wide association study (PWAS), Mendelian randomisation (MR), Summary-data-based Mendelian Randomisation (SMR), Protein-protein interaction (PPI), Heterogeneity in independent instruments (HEIDI), Transcription-wide structural equation modelling (T-SEM), Weighted gene co-expression network analysis (WCGNA), Phenome-wide association study (PheWAS), Polygenic risk score (PRS), Pharmacogenic enrichment scores (PES), Major depressive disorder (MDD), Cannabis-use disorder (CUD), Autism spectrum disorder (ASD), Bipolar disorder (BD), Schizophrenia (SCZ), Attention-deficit hyperactivity disorder (ADHD).

### Limitations and challenges

Despite the critical utility of TWAS/PWAS to integrate QTL data with GWAS for new genetic discoveries in psychiatry, there are several limitations (see [92] for a comprehensive outline). Some notable limitations include: 1) LD and gene co-regulation can obscure which gene is causal at a given locus; 2) genetically regulated expression models are limited by difficult-to-acquire post-mortem tissue sample sizes for psychiatric disorders; 3) TWAS does not factor in other modes of gene expression modulation, such as environmental factors or transcription factor regulation (this is also a strength of TWAS as associations cannot be explained by confounding).

Using MR to validate pleiotropic genes/proteins as drug targets for treating multiple conditions can be challenging. MR requires that the IV impacts the outcome only via the exposure (mediated/vertical pleiotropy) and not by other processes (horizontal pleiotropy). As these targets are identified as highly pleiotropic, careful selection of IVs associated with the phenotypes of interest is critical for MR analysis. However, the biological function of variants is often unknown, and the underlying mechanism of how a pleiotropic drug target is associated with each phenotype is difficult to pinpoint. Sensitivity analysis robust to pleiotropy must be performed (see [51, 93] for details), and IVs strongly associated with each outcome only through the exposure must be identified, e.g., via coloc [38] or HEIDI [94].

## Network computational drug repurposing

Network approaches can provide a broader understanding of the biological impact of pleiotropy. A pleiotropic component, such as a variant, gene, or protein, can affect many cellular functions through complex molecular interactions, causing possible pleiotropic effects [95]. Investigating these interactions using multiple layers of large-scale molecular information can inform the biological implications of pleiotropy in psychiatric disorders. Notably, using the appropriate methods to implicate genuine biological pleiotropy is essential. Like previous methods, molecular networks can uncover the physiological relevance of disease-associated variants and examine genotype-phenotype relationships to identify genetically informed drug targets for repurposing.

### Initial considerations

#### Data type

The central consideration for network methods is the data type used to build a network. Data can be sourced from several resources, including experimental data, text mining or databases. Drug repurposing studies often integrate multiple data sources to complete the missingness of each data level, an advantage of network approaches (refer to [96] for a comprehensive review of data sources and network-based drug repurposing for psychiatric disorders). See Table 4 for common data types and their uses.

**Table 4:** Common data sources to build disease-associated networks for drug repurposing and examples of their uses.

Data Sources	Network Components	Application Examples	
Genome	Disease-associated genes	• Typically integrated with other data layers to capture context-specific interactions of disease genes • Provide a foundation for investigating drug repurposing candidates from GWAS	
Interactome	Protein interactions Transcription factors Metabolites Gene regulators	• Maps functional connections between cellular components • Used to explore disease-specific contexts through integration with GWAS-linked genes • Determine additional drug targets through network propagation	
Transcriptome	Transcriptional profiles Transcriptional drug response	• Identify co-regulated genes and uncover putatively causal pathways • Infer molecular processes through latent mechanistic patterns • Predict protein interactions in different tissues and cell types	
Phenome	Drug indications Drug targets Drug adverse effects Disease symptoms Disease-associated targets Disease comorbidities	• Identify drug repurposing candidates through integration with drug targets and disease-associated genes • Examine the functional relationship between genes and diseases • Combine disease-genome and disease-phenome networks to explore shared mechanisms for reusable target inference • Predict drug adverse effects	
**Study Examples**	**Data Sources Integrated**	**Network Components**	**Drug Repurposing Findings**
Gao et al. [148]	Transcriptome Interactome Phenome	Transcriptional profiles Drug transcriptional profiles Protein interaction Drug-gene interactions	10 drug repurposing candidates for autism spectrum disorder based on network-specific core genes and opposition to disease transcriptional signature
Koch et al. [99]	Genome Interactome Transcriptome Phenome	Disease-associated genes Protein interactions Transcriptional profiles Transcriptional drug response Drug-gene interactions	12 drug repurposing candidates to treat the cognitive symptoms of schizophrenia via network propagation and opposition to the disease-gene expression profile
Truong et al. [149]	Transcriptome Interactome	Transcriptional profiles Transcription factors Protein interactions Drug transcriptional profiles	18 drug repurposing candidates likely targeting transcription factor regulatory pathways for the treatment of schizophrenia
Zhou et al. [150]	Genome Interactome Phenome	Drug adverse effects Drug-gene interactions Disease-associated genes Protein interactions	Five drug repurposing candidates are associated with increased odds of remission for opioid use disorders and could also be helpful for comorbid conditions

### Methodological approaches

**Fig. 6:**
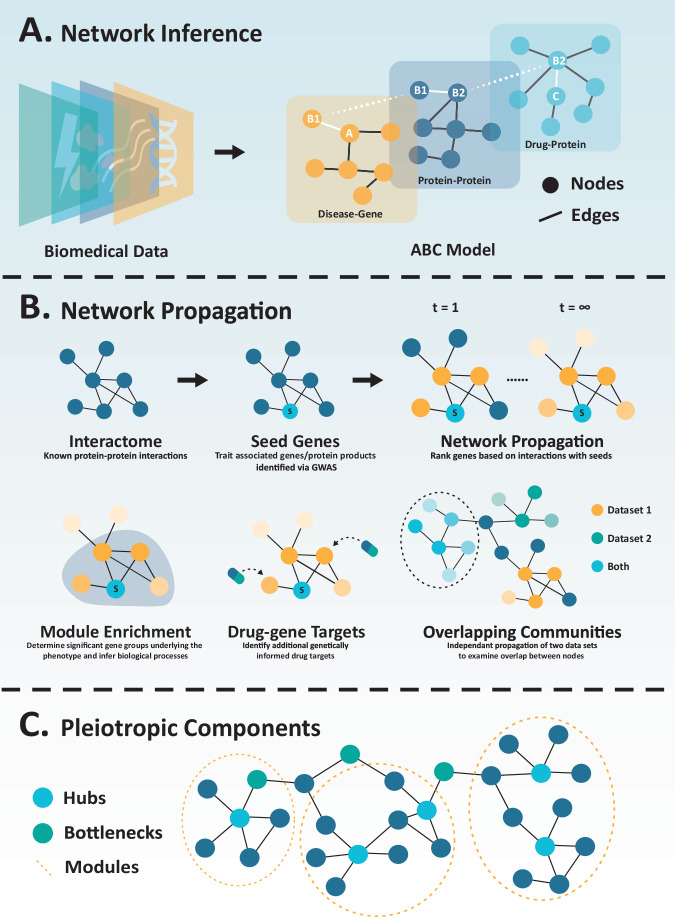
A network approach to unravel pleiotropy and identify genetically informed drug targets. **A** Using networks to explore biological connections. Constructing a network (network inference) involves integrating multiple layers of data to interpret relationships between biological components. The example demonstrates an ABC network model, connecting a disease gene to drug target. **B** Graphical representation of the process and applications of network propagation. Propagation can begin with integrating the known interactome with genome-wide association study (GWAS)-linked genes (seeds). Two time points of the propagation process are illustrated, with t = 1 demonstrating the initial state, and t = ∞ showing the final steady state (convergence). Network propagation can identify gene modules/clusters, additional drug targets, and overlapping communities between datasets. **C** Common pleiotropic properties of a network that may act as shared therapeutic targets.

The construction of a network (also known as network inference) involves using biomedical data to simplify the complexity of biological systems (Fig. 6A). The network nodes represent a system’s components, including genes, proteins, drug compounds, or diseases [96]. The interactions or relationships between nodes are represented as edges and inferred using physical connections, correlations, machine learning, or conditional dependencies (Fig. 6A).

#### ABC model

The ABC model is a classic network approach for identifying new links between components. The model assumes that if component A is linked to B and B is linked to C, components A and C are also linked. In this model, several steps (B_n_) can link A and C, and A and C must come from different data layers to generate latent connections. For example, used drug-gene networks (linking A to B) and disease-associated expression networks (linking B to C) to identify drug repurposing candidates.

#### GBA model

Guilt-by-association (GBA) is a method that relies on the connection between two entities in a network. It assumes that if two components are linked, they are more likely to share similar functions or be involved in related diseases. This approach uses similarity metrics to estimate new drug indications. If two diseases share significant characteristics, a drug for one disorder could be used for the other. Alternatively, if two drugs share the same properties, they are assumed to be useful for similar indications.

*Network propagation* is an application of guilt-by-association to predict additional disease-associated genes using GWAS and protein-interaction-based approaches (Fig. 6B). This is founded on the principle that interacting proteins contribute to similar biological functions, affecting the same trait [97]. Different algorithms can rank proteins as disease-associated based on their interaction profile with the disease-associated proteins identified via GWAS (*seed* proteins). Genes with a high frequency of interactions and thus shared function with a seed gene would be prioritised compared to those with low seed-gene interactions.

### Network pleiotropy and drug repurposing

#### Network propagation for unravelling pleiotropy and drug repurposing

Analysis of network-propagated gene interactions can determine highly connected modules enriched in disease association. These groups can infer biological mechanisms and shared pleiotropic cellular processes influencing disease. Network propagation has previously identified pleiotropic gene modules across GWAS traits [98] and pleiotropic drug targets shared between schizophrenia and cognitive performance [99].

Network propagation can also determine drug targets without genetic association to a disease. Many drug compounds with proven efficacy in clinical trials have targets without direct genetic evidence connecting them to the treated disease [4]. Many of these unsupported drug targets can be identified through network-based rankings, with studies demonstrating the enrichment of approved drug targets in genes that interact with disease-associated genes [100]. Expanding the interactome from a seed may reveal additional novel drug targets.

#### Targeting pleiotropic network components

Drug repurposing can leverage network-based methods to identify drug candidates targeting pleiotropic disease-associated components, such as specific nodes or clusters within a network (Fig. 6C). Investigating disease-network overlap from GWAS data can pinpoint shared disease components to provide a broader therapeutic effect. Common pleiotropic network components include:

##### Hubs

Pleiotropy has been linked to gene/protein network centrality [45]. The more central a gene or protein is within a network, the more likely it is to be involved in multiple processes [101, 102]. A central protein will have an increase in protein-protein interactions and higher distribution in cellular components, causing a protein’s molecular function to have a broader influence on biological processes [103]. Hubs are defined as having a high *degree* (i.e., number of connections), often in central network positions, and are integral topological components of a network.

Hubs are a common drug target in network medicine, as modulating the activity will have a more significant impact when compared to peripheral nodes [104]. Targeting shared hub nodes between diseases may have broad therapeutic benefits and treat multiple conditions due to their connectedness. For instance, one study identified five hub gene targets for potential drug targeting that were associated with both Alzheimer’s disease and major depressive disorder, two diseases with a high risk of comorbidity [105].

##### Bottlenecks

A pure bottleneck node is characterised by a low number of interactions, a high level of betweenness centrality (i.e., how central a node is), and often links subnetworks together [45]. Due to their strategic network positioning, these proteins can influence multiple diseases simultaneously, exerting their effects on distinct disease-related pathways. Many bottlenecks can also be classified as hubs if the node has many interactions and betweenness. These hub-bottleneck nodes are often evolutionary-constrained and associated with multiple pathways [106].

Bottlenecks and hub-bottleneck nodes are enriched in approved and experimental drug genetic targets, indicating betweenness as a promising measure for network drug repurposing [106]. Mahboubi et al. [107] identified a critical hub-bottleneck gene (*ESR1*) associated with both schizophrenia and obsessive-compulsive disorder (OCD) as an encouraging drug target for OCD-schizophrenia comorbidity [107].

##### Modules

Network modules are densely connected nodes with limited connections to outside nodes that may conduct a specific biological function [108]. Disease-associated genes tend to cluster together in well-defined neighbourhoods, known as disease modules, with a drug’s efficacy and potential adverse effects related to the proximity to these small network neighbourhoods [109, 110].

Menche et al. [111] observed overlapping network modules between disease pairs, with the extent of overlap strongly indicative of the pathobiological similarity between diseases. Shared modules between disease pairs are linked to genes demonstrating increased co-expression, common symptoms, and high rates of comorbidity [111]. Investigating module overlap between disorders can predict shared pathobiological functions for subsequent drug targets.

### Limitations and challenges

Network-based approaches in identifying novel drug candidates in psychiatry show much promise. However, there are still many challenges that must be addressed. First, current network-repurposing depictions only capture a fixed setting of the biological system, regardless of the dynamic nature of biological systems or the prevalence of transient interactions. Second, our knowledge of protein interactions is incomplete. The complexity of the interactome, including the vast number of proteins, their isoforms, and post-translational states, makes completing the interactome challenging. Third, networks are often based on known biological interactions and will be skewed towards well-researched mechanisms. Fourth, the direction of effect between network-associated gene interactions is usually unknown and requires further functional investigation. Fifth, establishing a causal relationship between disparities in gene expression and psychiatric disorders via analyses of transcriptomic networks can be difficult, as the disorder itself may induce changes in gene expression levels [13]. Lastly, integrating diverse data sources and managing heterogeneity can be complex due to sparse, incompatible, or absent data [112].

## Pleiotropy and adverse effects

Targeting a pleiotropic mechanism may result in a broader spectrum of therapeutic potential, but this will also increase the risk of adverse effects. Resources such as SIDER [113], a vast database on adverse drug reactions, would need to be included in any drug repurposing pipeline as a prudent measure. The genetic priority scores resource integrated SIDER, linking drug indications to genetic targets to develop a valuable tool for side effect prediction [48].

Genetic data can also be used to identify possible adverse effects when modulating a given target (see [114] for a full review). As previously described, PheWAS identifies multiple phenotypes associated with a variant. Alternatively, this method can predict potential adverse effects if a drug target is associated with other disease phenotypes in the corresponding direction of the drug mechanism, e.g., an agonist drug for a protein target that has a positive association with a disease phenotype. PheWAS has also been integrated into MR (MR-PheWAS) to examine the impact of exposure on numerous outcomes [115]. Similarly, this method can identify novel causal proteins/prioritising drug targets [116] and examine potential adverse effects [117]. This inference is based on an approved drug target (the exposure) being causal with outcomes other than its primary indication [118].

From a network perspective, targeting pleiotropic components may have safety-related limitations due to their broad influences. For example, targeting highly connected hubs may cause an increase in adverse effects or undermine network integrity [104]. Therefore, prioritising hubs with an intermediate degree can limit the risk of adverse effects. Alternatively, perturbing an interacting protein may modulate the effector protein to a lesser effect than a direct interaction or instead target the shared disease module the protein resides in. Drug-disease proximity is another important measure to predict a drug compound’s efficacy and detection of adverse effects, with a drug target required to be proximal to the disease module [110, 119].

Advances in technology and adverse effect datasets have enabled artificial intelligence, machine and deep learning methods to extract drug information and predict adverse reactions, e.g., via drug-to-drug networks [120] or neural networks and gene expression profiles [121]. Due to the expansive nature of pleiotropy, combining multiple approaches to predict adverse reactions when targeting such mechanisms is essential.

## Conclusions

Leveraging pleiotropy among psychiatric disorders may identify new treatments for comorbidities. This review outlines current research and methods used to investigate pleiotropy and identify drug repurposing opportunities for psychiatric disorders. Increasing sample sizes across psychiatric phenotypes will progress the identification of both shared and unique genetic risk factors. Alternatively, developing smaller but well-defined cohorts may be more helpful for drug repurposing, e.g., individuals with strict comorbidity diagnosis. This avenue of research would reduce the heterogeneity surrounding psychiatric disorders to identify specific biological mechanisms for precision medicine.

Future work should focus on combining evidence from multiple resources and methods to identify robust repurposing candidates for future validation studies. Multi-omic approaches are needed to gain a comprehensive biological understanding of genetic risk factors in psychiatric disorders for drug targeting. Developments in technologies and decreasing costs have improved the generation and feasibility of large-scale omics data, resulting in advances and prospects in multi-omic integration methods, such as machine learning models [122]. Additionally, electronic health records provide a valuable longitudinal and pathological patient data resource. Future access and incorporation of deidentified datasets into the drug repurposing process can assist in predicting and validating drug candidates [123].

In conclusion, understanding the underlying pleiotropic mechanisms across psychiatric disorders is a challenging task. However, improvements in data access, larger sample sizes, and advances in methodologies provide an excellent opportunity to continue our developments in unravelling the complex interplay across psychiatric genetic risk factors. The parallel between comorbidity and pleiotropy prevalence suggests shared genetic mechanisms that drug candidates could target to improve therapeutics for patients.
